# Drug resistance mediating *Plasmodium falciparum* polymorphisms and clinical presentations of parasitaemic children in Uganda

**DOI:** 10.1186/s12936-017-1777-0

**Published:** 2017-03-21

**Authors:** Stephen Tukwasibwe, Patrick Tumwebaze, Melissa Conrad, Emmanuel Arinaitwe, Moses R. Kamya, Grant Dorsey, Samuel L. Nsobya, Bryan Greenhouse, Philip J. Rosenthal

**Affiliations:** 1 Infectious Diseases Research Collaboration, Kampala, Uganda; 2 Department of Medicine, University of California, Box 0811, San Francisco, CA 94143 USA; 3 Department of Medicine, Makerere University College of Health Sciences, Kampala, Uganda

**Keywords:** Malaria, *Plasmodium falciparum*, Drug resistance, Virulence, Fitness, Uganda

## Abstract

**Background:**

*Plasmodium falciparum* genetic polymorphisms that mediate altered drug sensitivity may impact upon virulence. In a cross-sectional study, Ugandan children with infections mutant at *pfcrt* K76T, *pfmdr1* N86Y, or *pfmdr1* D1246Y had about one-fourth the odds of symptomatic malaria compared to those with infections with wild type (WT) sequences. However, results may have been confounded by greater likelihood in those with symptomatic disease of higher density mixed infections and/or recent prior treatment that selected for WT alleles.

**Methods:**

Polymorphisms in samples from paired episodes of asymptomatic and symptomatic parasitaemia in 114 subjects aged 4–11 years were followed longitudinally in Tororo District, Uganda. Paired episodes occurred within 3–12 months of each other and had no treatment for malaria in the prior 60 days. The prevalence of WT, mixed, and mutant alleles was determined using multiplex ligase detection reaction-fluorescent microsphere assays.

**Results:**

Considering paired episodes in the same subject, the odds of symptomatic malaria were lower for infections with mutant compared to WT or mixed sequence at N86Y (OR 0.26, 95% CI 0.09–0.79, p = 0.018), but not K76T or D1246Y. However, symptomatic episodes (which had higher densities) were more likely than asymptomatic to be mixed (for N86Y OR 2.0, 95% CI 1.04–4.0, p = 0.036). Excluding mixed infections, the odds of symptomatic malaria were lower for infections with mutant compared to WT sequence at N86Y (OR 0.33, 95% CI 0.11–0.98, p = 0.046), but not the other alleles. However, if mixed genotypes were grouped with mutants in this analysis or assuming that mixed infections consisted of 50% WT and 50% mutant genotypes, the odds of symptomatic infection did not differ between infections that were mutant or WT at the studied alleles.

**Conclusions:**

Although infections with only the mutant *pfmdr1* 86Y genotype were associated with symptomatic infection, this association could primarily be explained by greater parasite densities and therefore greater prevalence of mixed infections in symptomatic children. These results indicate limited association between the tested polymorphisms and risk of symptomatic disease and highlight the value of longitudinal studies for assessing associations between parasite factors and clinical outcomes.

## Background

Malaria, in particular infection caused by *Plasmodium falciparum*, remains an overwhelming problem in most of sub-Saharan Africa [1]. Malaria control is greatly limited by drug resistance. Resistance to a number of drugs is mediated in part by polymorphisms in two putative drug transporters, encoded by the *pfcrt* and *pfmdr1* genes [2]. The *pfcrt* 76T mutation is the major mediator of resistance to chloroquine and amodiaquine [3]. Polymorphisms in *pfmdr1* impact upon sensitivity to a number of drugs [4]. Considering polymorphisms that are common in Africa, the *pfcrt* 76T and *pfmdr1* 86Y and 1246Y mutations are associated with decreased sensitivity to chloroquine and amodiaquine, but these same mutations mediate increased sensitivity to lumefantrine and mefloquine [2, 5, 6].

The effects of drug resistance mediating genetic polymorphisms on the fitness and virulence of malaria parasites is of interest. Considering fitness, discontinuation of chloroquine for the treatment of malaria has led to dramatic changes in circulating parasites, with the return of chloroquine-sensitive *pfcrt* K76 wild type (WT) parasites and also of strong anti-malarial efficacy for chloroquine [7]. Clearly, chloroquine-sensitive parasites with WT sequence at *pfcrt* have a fitness advantage over resistant parasites. Considering *pfmdr1*, WT parasites outgrew those with three mutations, only one of which (1246Y) is common in Africa, in in vitro competition experiments [8]. Following mixed clinical isolates in culture over time, modest fitness advantages appeared associated with the mutant 86Y and WT D1246 alleles [9]. In an area with seasonal malaria, the prevalence of parasites with mutant *pfcrt* 76T and *pfmdr1* 86Y sequences decreased during the low transmission season, when drug pressure is lowest, implying a fitness advantage for WT parasites [10]. Overall, in most cases parasites with WT sequences in *pfcrt* and *pfmdr1* have appeared to have a fitness advantage over mutant parasites.

It is unclear if *P. falciparum* drug resistance-mediating polymorphisms also impact on parasite virulence, characterized as the ability to cause symptomatic or severe disease. Studies from India [11] and Mali [12], but not Sudan [13] or Gabon [14], showed associations between presence of the *pfcrt* 76T mutation and severe malaria, but these associations may have been due to clinical progression after chloroquine treatment failure, rather than variations in parasite virulence. Previously, differences in genotypes between parasites causing symptomatic and asymptomatic infections were studied in samples from a cross-sectional study of children from Tororo, Uganda. Remarkably, children harbouring isolates with mutations at *pfcrt* K76T, *pfmdr1* N86Y, or *pfmdr1* D1246Y had about one-fourth the odds of symptomatic malaria compared to those with WT parasites [15]. Similarly, in children in Benin, the prevalence of the *pfmdr1* 86Y mutation was significantly greater in asymptomatic compared to symptomatic infections, consistent with decreased virulence of parasites with the *pfmdr1* 86Y mutation [16]. However, the results of these case–control studies may have been confounded by two factors. First, infections associated with symptomatic disease typically have greater parasite densities than those that are asymptomatic, increasing the likelihood of detecting WT genotypes present in mixed infections. Second, children with symptomatic infection may have had a greater likelihood, compared to children presenting with asymptomatic infection, of a recent prior infection treated with artemether/lumefantrine, which selects for WT alleles [17–20]. To further explore relationships between parasite genotype and clinical presentation, samples were utilized from an ongoing longitudinal study and genotypes were compared for parasites in paired samples from Ugandan children with symptomatic and asymptomatic episodes of malaria.

## Methods

### Longitudinal study

Samples were collected from children enrolled in a cohort study in Nagongera Sub-county, Tororo District, Uganda, an area of high malaria transmission intensity. The cohort study has been described previously [21]. In brief, all households within the sub-county were enumerated and mapped, and 100 randomly selected households were enrolled if they met enrollment criteria of having at least one resident 0.5–10 years of age and at least one adult who provided informed consent. Subjects were encouraged to visit a dedicated study clinic open seven days a week for any illnesses. Febrile subjects (temperature ≥38 °C tympanic or history of fever in the past 48 h) were evaluated at the clinic, and if malaria was diagnosed based on any level of parasitaemia on a Giemsa-stained thick smear, treatment (artemether–lumefantrine for uncomplicated malaria; quinine for complicated malaria) was provided. Subjects also had routine blood smears obtained every 3 months. At the time of blood collection either for symptomatic disease or at routine visits, blood was also spotted onto filter paper and stored. The cohort study was approved by the Makerere University School of Medicine Research and Ethics Committee, the Uganda National Council for Science and Technology, and the University of California, San Francisco Committee on Human Research.

### Specimens evaluated for this study

For this sub-study, all subjects who met study criteria were identified. No a priori power calculation was performed for the sub-study. These subjects were aged 4–11 years and had two smear positive specimens, one from an episode of symptomatic (with fever, defined as above) malaria and another from an episode of asymptomatic parasitaemia, with each episode without prior treatment for malaria within 60 days, and the two episodes within 3–12 months of each other. These episodes all occurred between November, 2011 and December, 2014. Samples from each episode were collected and studied.

### Characterization of parasite polymorphisms

DNA was extracted from filter paper blood spots into 100 μl of water using Chelex-100 [22]. Gene fragments spanning all loci of interest were amplified, and multiplex ligase detection reaction-fluorescent microsphere assays were performed as previously described [20, 23].

### Statistical methods

Data analysis was done using R version 3.3 [24]. The primary outcome was presentation with symptomatic vs. asymptomatic malaria infection. Comparisons of the odds of genotypes present in symptomatic vs. asymptomatic paired isolates was performed grouping mixed infections as mutant, mixed infections as WT, excluding mixed infections from the analysis, or considering mixed samples as consisting of 50% WT and 50% mutant parasites. Analyses were performed using a binomial generalized estimating equations model, to allow for correlation between paired samples from each individual. A p value <0.05 was considered statistically significant.

## Results

### Characteristics of study subjects and episodes of malaria

Characteristics of the 114 subjects who provided samples for analysis and of the studied malaria infections are shown in Table 1. Similar numbers of children presented first with asymptomatic (54) compared to symptomatic (60) infections, and the mean age of the children when they presented with asymptomatic vs. symptomatic infections was very similar. The mean parasite density was about fourfold higher in symptomatic compared to asymptomatic infections.

**Table 1:** Characteristics of study children and paired malaria infections

Characteristic	Total episodes	Asymptomatic episodes	Symptomatic episodes
Number of episodes	228	114	114
Number with first presentation for each category	114	54	60
Age at time of sample collection (mean) (years)	7.00	7.02	6.98
Duration between episodes (mean) (days)	118	121	115
Parasite density (geometric mean)	9080/μl	3767/μl	14,390/μl

For each child, paired asymptomatic and symptomatic infections were studied, regardless of which presentation occurred first. For duration between episodes, the times shown are means for episodes preceded by a prior episode in the other category

### Prevalence of *Plasmodium falciparum* polymorphisms

Three *P. falciparum* polymorphisms that are associated with response to multiple anti-malarial drugs were studied [2]. All three of these mutations were previously very common in Ugandan isolates, but prevalence is decreasing, presumably due to decreased usage of chloroquine and increased usage of artemether–lumefantrine to treat malaria in Uganda [20, 24, 25]. Considering all study samples, about half were pure WT for the *pfmdr1* N86Y and D1246Y alleles, and <10% were pure WT at *pfcrt* K76T (Table 2).

**Table 2:** Prevalence of *P. falciparum* polymorphisms of interest in children with symptomatic and asymptomatic parasitaemia

SNP	Genotype	Total number (%)	Symptomatic (%)	Asymptomatic (%)
*pfmdr1* N86Y	Wild type	140 (64)	67 (61)	73 (66)
	Mixed	63 (29)	39 (35)	24 (22)
	Mutant	17 (7.7)	4 (4)	13 (12)
*pfmdr1* D1246Y	Wild type	98 (46)	47 (45)	51 (47)
	Mixed	69 (32)	38 (36)	31 (28)
	Mutant	48 (22)	20 (19)	28 (25)
*pfcrt* K76T	Wild type	14 (6.5)	7 (6)	7 (7)
	Mixed	8 (3.7)	6 (5)	2 (2)
	Mutant	193 (90)	99 (89)	94 (91)

Considering paired episodes in the same subject, thus controlling for host factors, the odds of symptomatic malaria were significantly lower for infections with mutant compared to WT or mixed sequence at N86Y (OR 0.26, 95% CI 0.09–0.79, p = 0.018), but not the other alleles (*pfmdr1* D1246Y OR 0.69, 95% CI 0.37–1.28, p = 0.24; *pfcrt* K76T OR 0.77, 95% CI 0.36–1.63, p = 0.49). However, if mixed genotypes were grouped with mutants in this analysis, the odds of symptomatic malaria were similar for mutant and mixed N86Y genotypes compared to pure WT (OR 1.23, 95% CI 0.72–2.10, p = 0.44). This difference was explained by the fact that symptomatic episodes were more likely than asymptomatic to be mixed (for N86Y OR 2.0, 95% CI 1.04–4.0, p = 0.036). Excluding mixed infections, the odds of symptomatic malaria were lower for infections with mutant compared to WT sequence at N86Y (OR 0.33, 95% CI 0.11–0.98, p = 0.046), but not the other alleles (*pfmdr1* D1246Y OR 0.82, 95% CI 0.46–1.43, p = 0.48; *pfcrt* K76T OR 1.08, 95% CI 0.43–2.75, p = 0.87). Assuming arbitrarily that mixed infections consisted of 50% WT and 50% mutant genotypes, the odds of symptomatic infection was 0.89 (95% CI 0.56–1.41, p = 0.61) for infections mutant at *pfmdr1* N86Y, 0.92 (95% CI 0.63–1.34, p = 0.66) for infections mutant at *pfmdr1* D1246Y, and 0.88 (95% CI 0.40–1.92, p = 0.75) for infections mutant at *pfcrt* K76T.

## Discussion

The prevalence of three *P. falciparum* polymorphisms associated with drug resistance was compared in children with either asymptomatic or symptomatic malaria infections in a cohort followed longitudinally in Tororo, Uganda. In 114 children with paired samples from episodes of asymptomatic and symptomatic malaria infections that occurred within a year, differences between genotypes of parasites from asymptomatic and symptomatic infections were much less marked than had been seen in prior case–control studies. One mutant genotype, *pfmdr1* 86Y, was more common in samples from asymptomatic compared to symptomatic infections, but symptomatic infections were more commonly mixed, potentially explaining the association. These results indicate limited association between the tested polymorphisms and clinical outcomes.

Decreased prevalence of *pfcrt* 76T and *pfmdr1* 86Y and 1246Y mutant genotypes in symptomatic, compared to asymptomatic infections was previously demonstrated in Ugandan children [15]. A study in Benin found a similar association for *pfmdr1* 86Y, but not *pfcrt* 76T [16]. These results suggested that these mutations render parasites less capable than WT parasites of causing clinical illness. However, the prior studies were case–control studies subject to confounding. First, children with symptomatic disease typically have greater parasite densities [15, 26], and thus a greater likelihood of detection of a mixed infection, than asymptomatic children. This was the case in the present study, with an odds ratio of 2.0 for mixed infections in symptomatic, compared to asymptomatic children. The increased prevalence of mixed infections likely at least partially explained the association between mutant genotypes and asymptomatic infection, as a greater likelihood of mixed infection would lead to a greater likelihood of WT parasites being identified in a symptomatic infection. In other words, symptomatic infections would be more likely than asymptomatic to have both WT and mutant parasites identified, thereby increasing the chance of WT being associated with symptomatic infections. Second, children with symptomatic malaria in cross-sectional evaluation were probably more likely to have had a recent prior episode of malaria, and thus more likely to have received recent treatment with artemether-lumefantrine, which selects for WT genotypes at the studied polymorphisms [17–20], and is the national first line regimen to treat malaria in Uganda and Benin [1]. In the present study, the influence of prior treatment was removed by studying only episodes without prior treatment for malaria within 60 days.

This study was designed to limit confounding in the comparison of parasites causing asymptomatic and symptomatic malaria infections. Utilizing samples from a longitudinal study, prior treatment histories were known, and only samples without prior therapy within 60 days, approximately the duration of selective pressure on the studied genotypes of artemether–lumefantrine, were studied [19]. To limit differences related to host immunity, only children over 4 years of age were studied; in this area of very high transmission intensity, children of this age would be expected to all have had numerous prior malaria exposures. To limit differences related to host factors, analysis of matched samples from the same patient was performed; roughly equal numbers of samples were from asymptomatic infections that occurred before or after symptomatic infections. With this study design, associations between parasite genotypes and clinical presentation were much less pronounced than seen previously in cross-sectional studies.

This study had some limitations. The sample size was necessarily limited by strict criteria for inclusion of samples for analysis, and so there may have been inadequate power to identify associations of small magnitude. The molecular assays had limited sensitivity, and so some minority genotypes that might play a role in disease presentation may have been missed. Of note, the molecular methods differed from those used in an earlier cross-sectional analysis in Uganda, but the two methods (restriction fragment length polymorphism analysis and multiplex ligase detection reaction-fluorescent microsphere analysis) have similar sensitivities [23]. Despite these limitations, use of longitudinal samples, with comparisons of samples from the same children, offered a valuable means of considering the impact of parasite genotypes on clinical presentation.

## Conclusions

Comparing two episodes of malaria parasitaemia in the same child with a design that limited influences of prior malaria treatment and varied prevalence of mixed infection, studied drug resistance genotypes had modest, if any influence on the risk of symptomatic malaria. Further studies of the impacts of parasite genetic polymorphisms on *P. falciparum* fitness and virulence are needed. These results highlight the value of longitudinal samples from well-studied subjects to answer questions concerning associations between parasite factors and clinical outcomes.
